# Supplementary material for: Are carbon emissions trading and green financial instruments synergistic? -Comprehensive quantitative research based on content analysis
Source: PLoS One. 2024 Mar 7;19(3):e0298601. doi: 10.1371/journal.pone.0298601 (PMC10919601; doi:10.1371/journal.pone.0298601)
Supplement: S1 Data — (ZIP) [file pone.0298601.s001.zip › Data/地级市Z_F工作报告.zip!/╜¡╬≈/╥╦┤║╩╨/└┤╫╘╣1⁄2╓┌║┼í╛┬φ┐╦╩2╛▌═°í┐_2015.docx]

各位代表：

　　现在，我代表市人民政府向大会报告工作，请予审议，并请各位政协委员和其他列席人员提出意见。

一、回顾去年，因地制宜，交出了一份成绩单

2014年，在省委、省政府和市委的正确领导下，全市上下围绕建设幸福宜春目标，紧扣“谋发展、比实干、争先进”要求，立足新常态，坚定抓项目，致力稳增长。预计全市生产总值1520亿元，增长9.8%；完成财政总收入272亿元，增长16.5%，其中公共财政预算收入190.3亿元，增长19.4%；500万元以上固定资产投资1356.2亿元，增长20.6%；规模以上工业增加值762.7亿元，增长12.4%；外贸出口22.3亿美元，增长21.6%；社会消费品零售总额457.3亿元，增长13%。

——发展升级有办法。产业升级方面：深入推进工业强市战略，总量首次进入全省前三强，其中非公经济贡献率达到95.6%，规模以上工业企业突破1000户，纳税超千万元企业246户；园区在建工业项目500余个、工业用电量突破100亿千瓦时、中国驰名商标数29件，均居全省第一；中石化宜春分公司成品油零售增幅列全国设区市第三；丰城再生铝、奉新棉纺织、上高绿色食品等10个列入省重点产业集群，宜春锂电、高安建陶、樟树医药进入省示范产业集群，数量均居全省第一。工业园区主营业务收入2900亿元，增长13.5%，其中在建、投产锂电企业49家，主营业务收入突破50亿元。奉新工业园完成扩区调区工作，丰樟高省级循环经济示范区建设扎实推进，上高工业园升格为重点省级园区，袁州、铜鼓省级工业园获批筹建，实现省级园区全覆盖。樟树获评全国中药原料生产供应保障、新型工业化中医药产业示范、盐化工循环产业三大基地，万载定为省LED照明灯饰产业基地，奉新荣获中国差别化纱线基地县，列入首批国家节能环保示范基地，靖安获得全省首个绿色低碳工业示范县。农业优势进一步巩固，完成总产值398.3亿元。粮食播种面积931.1万亩，总产415.4万吨；在全国率先整体推进基层国有粮食企业“退城进郊”工程，新增仓容12.9万吨，粮食托市收购量全省第一，市粮食局获评全国粮食系统先进集体。新建高标准农田6.6万亩，改造中低产田2.2万亩。粮食、畜禽、油茶、毛竹、苗木花卉、有机富硒等产业稳步发展，完成高产油茶造林11万亩，出栏肉牛28.4万头。新增市级以上农业龙头企业50家，达到330家。农民合作社突破4000家，居全省第三。宜丰荣获全国平安农机示范县，袁州获评全省休闲农业示范县（区），奉新连续六年获得全省农业综合开发先进县，高安连续五年居全省小农水重点县考核第一，樟树黄栀子获批国家地理标志保护产品。服务业快速发展，三年大会战有序推进，调度重点项目445个。接待游客2823.5万人次，旅游综合收入214亿元。明月山机场旅客吞吐量突破20万人次，创造了全省支线机场用时最短新纪录。成功举办第八届月亮文化旅游节、第二届万载国际花炮文化节、第九届奉新世界禅茶文化交流大会，泉月春晖——我为父母洗脚活动创吉尼斯世界纪录。禅博园开园接游，《禅境宜春》在中央电视台播出并获得第十届中美电影节最佳纪录片。明月山高山观光小火车、樟树古海度假区、万载烟花燃放国际赛事中心等投入运营。明月山创国家5A级景区通过专家评审，与台湾阿里山、日月潭结为友好姐妹山、友好景区，入选“2014中国旅游风云榜——‘美丽中国’十佳度假区”，奉新百丈山成功创新中国成立家4A级景区。温汤列为首批中国温泉旅游名镇试评示范镇，禅宗文化、明月山、靖安和樟树旅游产业集群列入省重点产业集群，靖安定为全省重点旅游县，宜春迎宾馆荣膺赣西首家五星级旅游饭店。世界500强家乐福集团进驻宜春，电商企业交易额突破百亿元，城市商业综合体项目和乡镇商贸中心建设数量均居全省第一。高安新增国家4A级物流企业7家，达到12家，居全国县级市第一。全市新增贷款突破180亿元，金融机构发展到17家，均居全省第三。丰城、高安、宜丰、万载农商行挂牌开业，总数达到8家，居全省第一。奉新中华包装在香港成功上市、小微企业信用体系建设经验向全省推广，宜春汽运公司成为首家“新三板”挂牌企业。区域升级方面：实施“双核两带”战略，发挥差异化考核激励作用，县域经济竞相发展，全省县域经济发展升级现场推进会在丰城召开。财政总收入超20亿元县市区6个、超15亿元8个，其中丰城居全国百强县第83位，较上年前移6位；樟树继续跻身全省十强县。市本级与“四区”管理权限进一步理顺，袁州干部职工“同城同酬”分步推进。与青海海东、福建莆田等地建立战略合作关系，袁州、樟树被纳入《赣闽粤原中央苏区振兴发展规划》。开放升级方面：坚持“走出去、请进来”，招大引强取得新突破。引进5000万元以上项目269个、亿元以上项目182个，分别居全省第三和第二，累计进资首超千亿元；利用外资5.9亿美元，增长10.9%。积极对接“央企入赣”，与正威国际、SK集团等世界500强和中节能、港中旅等央企签订合作协议。宜春海关获批筹建，高安、万载、樟树口岸作业区加快建设。举办美国烟花年会暨全国烟花展万载订货会。上高推进台资企业聚集工作法在全省推广，宜春检验检疫技术研究所获得全省首个进出口商品检验鉴定资质。获评全国贸促先进单位和2014浙商（省外）最佳投资城市，属全省唯一设区市。丰城、万载被评为全省十佳宜商县级城市，上高荣获全省首届最佳优化民营经济发展环境县。创新升级方面：与上海财大、上海股权托管中心等建立战略合作关系，与中科院、清华大学等合作共建院士工作站5家。新认定高新技术企业33家，总数突破100家，居全省第二；获批科技项目139项，专利申请量2800件，争取专利技术示范项目14个；首次获批省级小微企业创业园2个。 “四位一体，打造幸福工商联”做法获评全国工商联2014年度十大创新工作。袁州医药工业园定为省级高新技术产业化基地，丰城高新技术产业园获批省级孵化器和高新技术产业化基地，樟树、高安荣膺国家知识产权试点市，袁州、丰城、靖安、上高荣获全省知识产权富民强县示范县。节能减排工作扎实推进，市政中心、宜春职业技术学院荣获国家级节约型公共机构示范单位。

——小康提速有成效。民生支出占财政总支出比重超过70%。城镇居民人均可支配收入22854元，农村居民人均可支配收入10254元，分别增长9.5%和12.5%。市场物价稳定，居民消费价格指数涨幅控制在2%以内。新增城镇就业5.6万人，转移农村劳动力7.9万人。建成保障性住房20604套、农村危房改造7940户。打响城市棚户区改造三年攻坚战，开工建设项目80个，中心城区投资规模最大、拆迁面积最广、受益居民最多的黄颇路片区棚户区改造项目顺利启动，我市做法被中央电视台《新闻联播》专题报道。完成农村公路建设1592.1公里。建立失地农民社会保障制度，开展城乡低保专项清理整治，坚决纠正人情保、福利保。在全省率先出台彩票公益金助残项目救助办法，铜鼓下岗失业人员助保贷款工程在全省推广。樟树列入普惠型儿童福利制度建设全国试点，万载定为全国未成年人社会保护试点并在全国会议上作典型发言。在全省率先为8万计划生育家庭大龄独生子女购买团体保险。新改扩建城区中小学17所，增加学位4.6万个，教育“大班额”问题得到缓解。组建宜春幼儿师范高等专科学校，江西洪州职业学院一期建成并招生，靖安、铜鼓通过国家义务教育发展基本均衡县评估验收，宜丰、樟树荣获全省教育工作目标考核先进县。奉新支月英荣获全省龚全珍式好干部、方月萍当选全国十大最美村官，高安陈腊英获得全国最美乡村教师。荣登“中国好人榜”139人，居全国设区市之首，袁州“夺刀少年”柳艳兵、易政勇当选中央电视台2014年度法治人物。市人民医院（北院）、市儿童医院等项目顺利推进，中心城区古井保护得到加强。全省首部现代题材戏曲电影《阳台上的野菊花》成功首映并投放市场，原创廉政微电影《生日》获得第二届亚洲微电影艺术节“金海棠奖”，市文新局荣获全国文化系统先进集体。提前一年率先在全省实现县县有“三厅”以上数字影院。体育事业加快发展，在省第十四届运动会上再创佳绩。完善综治维稳经费保障机制，确保“三网”体系常态化运行，平安宜春建设稳步推进，被交通运输部定为平安交通试点市。第九届村（社区）“两委”换届选举有序实施。市应急救援综合保障基地开工，樟树应急救援大队被省政府授予模范大队。安全生产态势总体平稳。

——绿色崛起有保障。牢固树立绿水青山就是金山银山的理念，以生态文明建设促进经济社会发展。设施改善。深入推进城建三年升级战，全市开工项目722个，累计完成投资805亿元。杭长高铁通车，新火车站综合交通枢纽工程运营，环城南路贯通，320国道绕城改建工程开工，数字城管平台初步建成，公共自行车免费租赁系统抓紧启动，4万吨/日污水处理厂扩建、50吨/日污泥无害化处理等项目加快推进。宜春入选全国20个生活最幸福城市和全省首批生态宜居城市，樟树获批国家新型城镇化试点县（市），樟树、奉新、铜鼓荣获江西十大最具幸福感城市，万载获评最具魅力节庆城市。面貌改观。按照“当县建镇”思路，19个示范镇（重点镇）镇村联动建设扎实推进，“新建二十个一、五个提质改造”项目基本完工。每个示范镇（重点镇）增设城管中队、交警中队、环卫所、规划所等机构，管理功能更加完备，我市做法在全省现场会上作典型发言。丰城曲江、万载株潭、靖安宝峰等14个入选全国重点镇，高安八景、铜鼓大塅、袁州西村等15个入选全省百强中心镇，数量均居全省前列。市本级和高安被评为全省耕地保护成效显著设区市、县，经验做法被中央电视台报道。在全省率先启动改善农村人居环境，实施“两清一通”工程，清理农村垃圾46万吨，农村环境有效改善。新增国家、省级生态乡镇29个，铜鼓通过国家级生态县技术评估，靖安定为全国生态文明示范工程试点县，其城乡垃圾一体化处理模式在全省推广。治理改进。联合开展环保专项行动，查处违法违规企业197家，停产治理32家，取缔关闭13家。加大生猪养殖、水库水质污染治理，中心城区饮用水源地保护区养殖场全部关闭。出台“新锦江十条”，环境综合整治成效初显，得到省领导高度肯定，并在全省推广。昌铜高速生态经济带列入国家发改委等部委批复的省实施方案。农产品品牌和地理标志达到643个，奉新获评中国绿色生态示范县、猕猴桃示范县和全国森林公安严打工作先进单位，丰城生态硒谷定为首批国家科技特派员创业基地，上高获得全国绿色生态农业示范县、苗木发展示范县，铜鼓荣获中国竹键盘之都。

——实干兴宜有定力。促项目。完善重大项目协调推进机制，召开政法军队口、丰城、袁上万片区等现场会，加快项目落地。重大项目在库总数373个，其中10亿元以上项目157个，全面竣工投产50个。丰城龙头山水电航运枢纽工程、靖安洪屏抽水蓄能电站等项目加快推进，万宜高速基本建成，昌樟高速改扩建和昌栗、昌宁以及东昌高速公路建设有序推进，丰城至南昌一级公路贯通。抓改革。按照“1+N”的思路，出台改革文件38个。进一步简政放权，落实行政审批事项236项，精简市级审批事项667项，精简率达57.1%。组建行政审批中介服务“超市”，进驻涉审中介机构40余家。实施注册资本登记改革，新登记企业数、注册资本总额分别增长72.1%、131.4%。加大市直国有资产整合力度，重点打造发展投资集团等四大平台。支持丰城开展省直管县（市）体制改革试点，下放经济社会管理权限729项。上高撤县设市工作有序推进，丰城董家被中央编办确定为全省3个经济发达镇行政管理体制改革试点镇之一。率先全省启动农村土地确权工作，铜鼓颁发全省土地承包经营权“第一证”，并在改革工作推进会上作典型发言。不断规范土地流转，率先全省建成市、县、乡、村四级服务平台，在全国会议上作经验交流。58个国有林场改革全面完成。转作风。深入开展党的群众路线教育实践活动，明确整改事项176项，办结154项，办结率达87.5%。认真落实党风廉政建设责任制，严格执行中央“八项规定”，“三公”经费明显下降。扎实推进法治政府建设，重新修订政府工作规则，出台重大行政决策听证办法，实施机构编制动态管理，在全省率先建立“全口径四大预算”体系上人代会审议机制。全面推广奉新经验，普遍建立法律顾问制度的做法得到中央领导肯定。稳步推进“六五”普法，奉新获评全国“六五”普法中期先进县。监察、审计工作进一步加强，信访工作保持全省前列。认真执行市人大及其常委会的决议、决定，自觉接受市人大、政协和社会各界监督，办结市人大代表建议和政协委员提案313件，荣获省第十二届人大代表建议办理工作先进单位。加大信息公开力度，主动公开政务信息11.5万条，组织在线访谈14期，召开新闻发布会18场。与此同时，国防后备力量建设、食品药品监管、国家安全、外事侨务、民族宗教、涉台事务、邮政通信、烟草、盐业、气象水文、防震减灾、无线电管理、妇女儿童、统计、档案、扶贫、老龄和红十字会等工作均取得新成效。

各位代表，一年来，市政府紧紧依靠市委的统揽指挥，紧紧依靠市人大、市政协的监督支持，借天时，扬地利，聚人和，用更多的心血和汗水，尽最大的努力和智慧，巩固了发展的好势头，激发出巨大的正能量，推动宜春站上了更高发展平台。这些成绩的取得，是各级领导班子团结协作的结果，是全市上下奋力拼搏的结果。在此，我代表市人民政府，向付出辛勤劳动的全市人民，向支持政府工作的人大代表和政协委员，向各民主党派、工商联、无党派、人民团体、驻宜部队、民兵预备役、武警官兵、公安民警和社会各界人士，向所有关心支持宜春发展的同志们、朋友们，致以崇高的敬意和衷心的感谢！

前进道路上，我们清醒地认识到，还存在着一些深层次的矛盾和问题：经济增长动力不足，企业数量少，体量不大，抗风险能力弱，部分项目落地难、推进慢、见效差，高新技术产业引进少、贡献小；农业产业化水平不高，示范园区辐射带动不强；城乡一体化进程推进不够，城市基础设施有待完善，停车难、交通拥堵等问题亟需缓解；社会事业欠账较多，安全稳定任务繁重；行政效能不够高，不作为、乱作为的现象依然存在，等等。对这些问题，我们将采取有效措施克服解决，决不辜负全市人民的厚望和重托！

二、谋划今年，冷静分析，明确了一张时刻表

各位代表，我们时刻谨记，新一届政府组建以来，肩负三大使命：改善条件，为发展增添动力；做大总量，为升级赢得空间；兜底民生，为百姓提供保障。通过几年来的磨合与实践，三项使命取得进展。当前，本届政府步入届中的关键期，宜春迎来“海陆空”新时代，中心城“三个中心”的定位不断深化：由于历史的原因，在宜春不是中心，在赣西是中心；随着交通的改善，在江西不是中心，在中部是中心；通过不懈的努力，现在可能不是中心，今后肯定会是中心。基于对定位的理解，中心城的建设和管理要赋予新的理念。身处赣西区域中心，竞争与合作并存，做大总量、融入区域迎来新机遇，赣西三市首次合作大会在宜举办，一批项目扎实推进，为西翼腾飞插上坚强的翅膀；身处中部崛起战略中心，长江经济带、鄱阳湖生态经济区等重大战略的实施，承东启西的优势更加明显，有利于争取更多的支持，四方井水利枢纽工程顺利推进，圆了宜春人30年的梦想，蒙西至华中铁路煤运通道、昌吉赣客专动工，穿越宜春多个县市；作为全市政治、经济、文化中心，政策、资金、人才等要素加快涌入，投资、消费更加活跃，开放的层次不断提升，“虹吸效应”开始显现。处在这样伟大的时代，参与这场生动的改革实践，与其说是我们的努力，不如说是我们的幸运。面对组织的重托、人民的期待，我们心怀十分的感恩，仍需付出百倍的努力！

2015年是全面深化改革的关键之年、全面推进依法治国的开局之年，也是全面完成“十二五”规划的收官之年。综观大势，经济增速“七上八下”成为新常态，实体经济不振，人口红利消退，“刘易斯拐点”带来挑战。我们既要“爬坡过坎”，又要“抬头赶路”，任务繁重，机遇难得：中央全面深化改革和依法治国重大部署协同推进，内生活力不断释放，新增动力正在生成；省委、省政府把定航线，保持发展、战略、实干三大定力，重点谋划产业集群、县域经济，发展布局基本完成；市委围绕全面建成小康社会，坚持一张蓝图绘到底，全市上下谋发展的氛围更加浓厚、比实干的斗志更加昂扬、争先进的意识更加强烈，发展气势已经形成。我们要抢抓机遇、顺势作为，经略周边、协作融合，在新一轮发展升级大潮中争得先机、赢得主动。

今年政府工作的总体要求是：全面贯彻落实党的十八大、十八届三中、四中全会和中央、省经济工作会议精神，紧扣省委“十六字”方针，坚持稳中求进工作总基调，主动适应新常态，突出传统产业升级，发展高新产业，壮大县域经济实力，把经济转型升级、提质增效放在更重要位置，同步推进改革开放与法治建设，提高生态建设水平，增进民生福祉，保持社会和谐稳定，为与全省全国同步全面建成小康社会奠定坚实基础。

全市经济社会发展主要预期目标为：生产总值增长9.5%左右；财政总收入增长15%，其中公共财政预算收入增长15%；规模以上工业增加值增长11.5%；500万元以上固定资产投资增长19%；社会消费品零售总额增长 12.8%；实际利用外资增长11%，外贸出口增长7.5%；城镇居民人均可支配收入增长10%左右，农村居民人均可支配收入增长11.5%；城镇化率提高2个百分点；城镇登记失业率控制在4.5%以内，人口自然增长率控制在 8 ‰以内，居民消费价格指数涨幅控制在3%以内。

各位代表，上述目标结合了上级的部署要求，契合了宜春的具体实际，只要我们精诚团结、开拓进取，一定会圆满实现！

三、突出重点，精准发力，制定了一本任务书

新的一年，我们将聚焦发展升级，聚力项目建设，聚神操作落实，蓄跨越之势，鼓实干之劲，朝着全面建成小康宜春的幸福大门奋力迈进。重点抓好六个方面：

（一）引领新常态，加速产业升级的步伐

以提升产业竞争力为核心，重点培育龙头企业，强化要素配套，谱写产业升级新乐章。

工业做强。以加速推进产业集群为抓手，推动优势企业、产业、资源和生产要素向工业园区集中，完善上下游协作配套，提升产业竞争力。推进一批省级重点产业集群、壮大一批市级重点产业集群、培育一批地方后备产业集群，改造提升传统产业，积极发展新兴产业，筛选200户企业重点培育，力争规模以上工业企业新增120户、纳税超千万元企业达到260户，工业园区主营业务收入3400亿元，十大省级重点产业集群主营业务收入1350亿元，打造百亿产业集群8个。

农业做特。围绕把宜春建设成为全省现代农业先行区、农村改革探索区、农村清洁工程示范区和全国绿色有机农产品生产基地的目标，继续发展粮食、畜禽等六大优势产业，重点发展油茶、肉牛、绿色有机、富硒和生态休闲农业，着力打造靖奉宜铜万有机农业带、丰樟高富硒产业带、袁靖铜休闲农业示范区和上高富硒功能农业区。加快现代农业示范园区建设，支持创建现代农业科技博览园，袁州、樟树申报国家级现代农业示范区，万载筹建有机产品检测区域分中心，争取万载获评全国有机产品认证示范县。积极培育新型经营主体，力争农民合作社、家庭农场分别突破4500家、800家。实施大中型灌区续建配套与节水改造，抓好小农水重点县建设。加强耕地保护，坚持依法依规、节约集约用地，开展闲置低效用地清理，盘活闲置用地1.3万亩以上。推进实施 “万村千乡市场工程”和基层国有粮食企业“退城进郊”工程，力争70个新扩建库点开工。

服务业做响。按照“双核三线四游”的总体布局，整合传统与新兴媒体，加大“一年四季在宜春”的宣传，突出智慧旅游营销，拓展中远程客源市场，促进旅游产品向观光、休闲、度假并重转变，吸引更多游客来宜“春看花、夏避暑、秋赏月、冬泡泉”，力争接待游客突破3500万人次、旅游综合收入超270亿元。争取明月山创国家5A级景区成功摘牌，完成禅宗文化26集动画片制作和发行，筹办好第九届月亮文化旅游节。推进宜春禅宗文化、明月山、靖安和樟树四大旅游产业集群发展，加快新火车站综合交通枢纽游客集散中心、明月山温泉文化带旅游综合体和月之泉·国际硒温泉康疗中心、袁州西海温泉、樟树开元名都大酒店、奉新九仙温泉等重大旅游项目建设，支持靖安三爪仑旅游景区、明月山温汤分别争创国家5A级和世界温泉名镇。深度挖掘禅宗文化内涵，加强海峡两岸禅宗文化旅游交流。创建一批省级电子商务示范基地和企业，推进电子商务进农村示范建设，争取电商交易额突破150亿元。加大“财园信贷通”、“财政惠农信贷通”、“银园保”、企业信用互助等融资力度，探索土地承包经营权抵押贷款，创优文化旅游产业融资模式，力争新增贷款180亿元、引进外埠银行1家。引导民间资本设立投融资公司和电子商贸公司，推进小额贷款和融资担保公司规范经营。鼓励企业境内外上市，争取成功上市1家、“新三板”挂牌3家。

各位代表，产业支撑不强是宜春的劣势，我们将强化重点产业对接，全面提升竞合能力，为发展注入源源不断的动力！

（二）加强微刺激，巩固县域经济的优势

兴市之基在于强县，强县之路在于发展。进一步松绑给力、创优环境，加快县域经济块状隆起，为发展升级稳固基石。

东西“双核”驱动。深化“双核两带”发展战略，做强丰樟高循环经济板块，叫响袁上万城市经济板块，打造以靖奉宜铜为核心的生态经济带。丰樟高“金三角”要有争当全省“排头兵”的底气，继续保持领先位置，利用毗邻南昌的地缘优势，积极融入南昌大都市圈，争取在基础设施配套、产业转移承接等方面获取更多支持，进一步提升产业核心竞争力，形成循环经济隆起带，成为对接长江经济带、鄱阳湖生态经济区的“桥头堡”。袁上万“新三驾马车”要有跟进追赶的勇气，高举“做强袁上万、比拼丰樟高”的大旗，以320国道为“轴线”，科学编制区域规划，合理确定发展规模和空间布局，促进资金、技术、产业等互动交流，推动企业、产业横向兼并重组，打造县域经济新增长极。靖奉宜铜“四小虎”要有不甘落后、自我发展的锐气，抓住交通大改善的机遇，发挥后发优势，加快发展旅游养生业，适度发展新型工业，做大经济总量，做优生态环境，走出一条经济发展与生态保护共赢的特色之路。

产业集聚拉动。立足自身优势，明确产业布局，各地重点培育2-3个产业集群，加快将建材、机电、食品、医药、化工等打造成千亿产业，市经开区、丰城高新技术产业园及樟树、高安、上高、奉新工业园等发展成千亿园区，四特集团、济民可信、仁和集团、奥其斯、旺旺食品、睿锋环保、金纶集团等培育成百亿企业。市经开区主抓雄塑科技、宜春客车厂、六星集团等项目，袁州主抓科伦、红狮水泥、莱茵电梯等项目，樟树主抓宏宇能源、金属家具产业科技园、医药产业物流园等项目，丰城主抓华伍制动、唯美陶瓷三期、格林美三期等项目，靖安主抓连天红家居产业园、东白源道教养生谷、香樟林养生谷等项目，奉新主抓泰明光伏、中江纺织、洁彩纺等项目，高安主抓中节能、中汽零、康铭盛等项目，上高主抓赣西电商谷、华西希望·四川特驱、金利隆橡胶履带等项目，宜丰主抓景田百岁山、鼎盛玻璃、旭锂矿业等项目，铜鼓主抓汤里温泉、奔步科技、昆庆纺织等项目，万载主抓志成实业、万佳通照明灯饰、恒晖农业等项目。通过项目建设，拉动产业集聚，壮大经济总量。

市县上下联动。推进扩权强县，抓好丰城省直管县（市）体制改革试点，支持上高撤县设市，赋予县市区更大自主发展权。进一步完善差异化考核制度，引导各地不比条件比干劲，不比规模比质量，不比现状比势头，营造竞相比拼、奋力赶超的良好氛围，争取经济总量处于全省第一方阵县市区前移1位、第二方阵及其后县市区前移2位、丰城在全国百强县排名再进位。放手支持袁州与“三区”做大做强，两年内实现袁州干部职工“同城同酬”。强化对重大项目的跟踪调度，做好协调服务，组织召开丰樟高、靖奉宜铜片区和“三区”重大项目现场推进会，破解项目瓶颈，增强发展动力。

各位代表，县域经济是宜春跨越崛起的筋骨，我们将因势利导，突出重点产业，提升发展层次，再创县域经济新辉煌！

（三）留得住乡愁，提升统筹发展的水平

坚持扩容与提质并重，以镇村联动建设为抓手，积极探索城镇发展新路，让城乡居民望得见山、看得见水、留得住乡愁。

关于中心城区。中心城区的发展，亮点在新城，重点在强城，难点在老城。大力发展现代物流、电子商务、商贸服务等新兴产业，进一步优化人居环境、完善城市功能，集聚人气、财气、商气。积极对接新宜萍城镇密集带，加快机场片区、高铁片区和明月大道、环城南路两侧开发，抓好320国道绕城改建、四方井水利枢纽工程建设和宜新、宜慈公路改造提升，推进北湖公园、北岸新天地、百尚城、红林世界城等项目建设，加快张家山汽车物流城、大塘商业街、昌黎北路延伸、跨高速公路规划路建设，争取赣西万达项目落户。深入实施城市棚户区改造三年攻坚，突出抓好黄颇路片区和李家组、秀江东路延伸片区等城市棚户区及背街小巷改造，完善老城区排水管网设施，让群众从“忧居”变“宜居”。适时启动飞剑潭水库备用水源建设，统筹解决中心城区及沿途乡镇用水安全问题。规范房地产市场秩序，促进平稳健康发展。

关于镇村联动。进一步完善镇村功能，提升集镇管理水平。全面完成首批19个示范镇（重点镇）试点，启动第二批示范镇（重点镇）建设，原则上每个县市区、明月山申报1个，争取打造一批工业强镇、商贸重镇和旅游名镇。大力推进中心村建设，增强公共服务辐射力和经济发展带动力，促进镇村道路、供水排水、供电通讯等基础设施共建共享。加快农村人居环境改善，建立垃圾处理长效机制，实现看得见、可持续的变化。

关于基础设施。主动策应“一带一路”、长江经济带、苏区振兴发展、鄱阳湖生态经济区等战略，谋划和包装一批重大项目，力争挤进国家和省“十三五”规划“笼子”。做好昌樟高速公路改扩建扫尾，加快推进蒙西至华中铁路煤运通道、昌吉赣客专、昌栗、昌宁、铜万、东昌高速公路等项目建设，抓好奉新鹅婆岭水利枢纽和抽水蓄能电站、丰城龙头山水电航运枢纽工程和丰电三期、靖安洪屏抽水蓄能电站二期、上高大唐国际电厂、奉新—高安—昌傅高速公路等项目前期工作。加快干线公路建设，力争完成县乡道升级改造120公里、客运网络路建设260公里、自然村通水泥路1000公里。

各位代表，新型城镇化贵在突出“新”字、核心是写好“人”字，我们要努力消除城乡差异，实现农村与城市共同繁荣！

（四）亮出“三清单”，激活改革创新的活力

改革是开路先锋。落实深化改革“规定动作”，探索宜春改革“自选动作”，不断释放改革新红利，增添创新原动力。

深化改革。加快政府职能转变和机构改革，全面实施“三单一网”管理，拿出“权力清单”，做到“法无授权不可为”；给出“负面清单”，做到“法无禁止皆可为”；理出“责任清单”，做到“法定职责必须为”，进一步完善政务便民服务网。衔接国务院、省政府取消和下放的审批事项，加强事中事后综合监管。推进事业单位分类改革，实现政事分离、政企分开。深化财政体制改革，加快构建“全口径四大预算”体系。加大国有资产整合，确保发展投资集团良性运转，同步建成旅游、公路、城建三大发展平台。在推进农村承包地、宅基地等确权登记颁证基础上，加快农村集体产权、水利产权制度改革，建立现代农村产权制度。健全农村土地流转服务平台，引导经营权规范有序流转，发展农业适度规模经营。遵循城镇化发展规律，顺应人民群众意愿，加快推进户籍制度改革。抓好国企改革扫尾工作，妥善解决宜工机械、长青厂、房地产开发总公司等改制遗留问题。实施教育综合改革，推进义务教育免试就近入学、九年一贯制对口招生和校长教师交流轮岗。深化公立医院改革，优化医疗资源配置，鼓励社会办医。开展公办养老机构改革试点，推广公建民营、民办公助养老服务模式。

扩大开放。突出招大引强，加快对接频率，紧盯世界500强和重大央企，力争引进亿元以上重大项目200个、30亿元以上项目6个、央企项目10个，资金超490亿元。主动跟进新签约的市经开区睿泰涂布、樟树为诚医药、丰城东江环保、上高如益科技、万载睿达新能源、奉新陆丰管业等超10亿元项目，确保落得下、建设快。实施宜商回归创业工程，吸引更多在外成功人士返乡投资创业。争取启动海关大楼建设，明年开关运营。推进高安、万载、樟树口岸作业区建设，开通宜春至深圳或宁波集装箱铁海联运阶梯式班列和九江港烟花爆竹运输通道，完善宜丰康替龙竹业集装箱国检监管区服务功能。优化出口产品结构，鼓励企业到境外从事矿产资源、农业投资开发和营销网络建设，拓展建筑陶瓷、金属家具产业国际市场。

创新驱动。深入实施科技创新“六个一”工程，力争高新技术产业增加值达 150 亿元。新增中国驰名商标 5件，申请专利突破3000件，促成200个产学研科技合作项目落地，列入国家科技支撑计划和省战略性新兴产业引导资金项目 7 个，获得省政府科学技术奖励科技成果6项，争取华伍制动工程技术研究中心升格为国家级。推进国家陶瓷产品质量监督检验宜春分中心和省级锂电产品质检中心建设，组建省级研究中心 2 个。加强特色新型智库建设，完善招贤引智机制。加大小微企业支持力度，争取新增小微企业3000家。营造鼓励探索、允许失误、宽容失败的干事创业环境，形成“人人创新、万众创业”新局面。

各位代表，改革只有进行时没有完成时，我们一定管住约束“到处插手”，充分发挥市场“调节之手”，用政府的“减法”换取市场的“加法”！

（五）坚守生命线，保护绿水青山的环境

以全省建设生态文明先行示范区为契机，全面落实“新锦江十条”，切实做到源头严防、过程严管、后果严惩，探索可复制、可推广的宜春模式。

重拳抓治理。认真贯彻新《环境保护法》，对环境违法行为“零容忍”、出重拳、用重典，让排污企业不能违法、不敢违法、不想违法。抓好工业、建筑等重点领域节能降耗，化解水泥、陶瓷等行业产能过剩，淘汰一批落后产能企业，单位工业增加值能耗下降3%以上。完善污水处理设施及配套管网建设，启动垃圾综合处理场建设，加快淘汰“黄标车”，强化陶瓷、重金属、生猪养殖和农业面源污染防治，实现所有小（二）型以上水库养殖“人放天养”。总结提升锦江经验，加强袁河、潦河等重点流域污染治理。

齐心促创建。大力实施生态修复工程，推进昌铜高速生态经济带建设，突出抓好复绿治污、封山育林等工作，提升森林生态功能等级，引领全省生态文明示范区创建。加强自然保护区、湿地公园、森林公园、水功能区、风景名胜区等重要生态功能区域保护，加快国家生态园林城和森林城市创建，支持铜鼓、宜丰创新中国成立家级生态县，奉新、上高创新中国成立家园林县城，袁州、丰城、奉新、万载创建省级生态县，着力打造一批国家、省级名片，提升宜春生态美誉。

合力打基础。秉承“绿色、低碳、循环”发展理念，加快中国生态硒谷现代产业园、上高绿色食品产业园、万载有机食品加工基地、樟树农业“两区”建设，构建绿色工业、生态农业和现代服务业为主的绿色产业体系。积极创建全国低碳试点城市，支持丰城申报国家城市矿产示范基地，加快奉新国家节能环保示范基地建设，推进丰樟高创建全省循环经济示范区，鼓励袁州、靖安发展低碳绿色产业，着力打造煤电、盐化工、废旧金属及塑料回收等循环产业。促进新能源汽车推广应用，全面完成省下达的指标任务。

各位代表，生态是宜春最大优势、最大品牌，我们将以刚性制度和严厉举措，让宜春山更清、水更秀、天更蓝、城更美！

（六）共筑宜春梦，增进社会民生的福祉

统筹安排，整体推进，持续加大民生投入，让全市人民更多更公平地分享改革发展成果。

繁荣各项事业。实施第二轮学前教育三年行动计划、普通高中资源扩充工程和农村义务教育学校标准化建设，新改扩建城区中小学18所。提升宜春学院、宜春职业技术学院、宜春幼儿师范高等专科学校内涵发展水平。推进文化惠民工程，全面实施广播电视“户户通”。争取禅宗文化内涵项目申报国家级非物质文化遗产。配合做好第一次全国可移动文物普查和第二次全国地名普查。加快市人民医院（北院）等项目建设，完成中心城区古井保护工程，建立公共自行车免费租赁系统，拓展金融IC卡在公共服务领域应用，推进数字档案馆、图书馆建设。健全医疗卫生服务和基本药物供应保障体系，完善流动人口计划生育信息共享和公共服务机制，促进人口计生长期均衡发展。加快市、县、乡、村全民健身服务网络建设，重视竞技体育后备人才培养。

改善民生保障。整合城乡居民基本医疗保险制度，扩大社会保险覆盖面，完善社会养老服务体系。加快市残疾人康复托养中心建设，推广残疾人微创业和居家就业经验。鼓励创业带动就业，力争新增城镇就业4.7万人，转移农村劳动力6.7万人。推进保障性安居工程建设，妥善解决中心城区房屋产权登记遗留问题。优先发展公共交通，有效缓解交通拥堵及停车难。落实城乡困难群体帮扶救助政策，做好“雨露计划”培训、移民搬迁、村庄整治和产业扶贫。推动“菜篮子”工程建设，抓好蔬菜基地、直销店、农超对接工作。实施农村安全饮水工程，解决27万人口饮水安全问题。

创新社会管理。推广应用“三网”体系，从严打击各类违法犯罪活动，不断提升公众安全感和满意度。健全法律援助制度，重视群众来信来访，帮助解决合理诉求。切实做好征地拆迁补偿安置工作，规范失地农民养老保险。落实“党政同责，一岗双责”制度，深化安全生产专项整治，防范较大以上安全事故发生。加快食品药品检测检验中心建设，建立全程监管体系，确保“舌尖上的安全”。引导群众理性投资，健全债务预警机制，有效防范金融风险。推进应急救助平台建设，增强综合防灾减灾救灾能力。完善妇女之家建设和管理办法，提升留守儿童、留守妇女、留守老人关爱服务水平。加强国防动员、民兵预备役、人民防空建设，加快双拥模范城创建。做好民族宗教工作。

各位代表，政府自身建设永远在路上。我们将恪尽职守、夙夜在公，不断提升学习力、执行力、创造力，加强法治和服务型政府建设。更加依法规范。全面推进依法行政，牢记权力法定意识，规范行政权力运行，完善政府法律顾问制度，确保依法用权、秉公用权、为民用权。做好人大代表建议、政协委员提案办理。落实党风廉政建设责任制，恪守中央“八项规定”和国务院“约法三章”，坚决反对和纠正“四风”，加强项目招投标等重点领域行政监察和重点民生工程等执行情况审计监督。更加务实担当。强化谋大事、干实事意识，激活红色基因，弘扬优良传统，争做有信仰、有担当、有情怀、有气节的好党员、好干部，敢于担责，敢打硬仗，接受任务不讲条件，落实任务不找借口，完成任务不打折扣。更加关注民生。大力压缩“三公”经费支出，真正做到“政府过紧日子，人民过好日子”。去年重点实施了“10+1”工程，132个子项目达到或超过进度，16个因土地、资金及政策调整等原因未达进度要求。今年突出抓好“十大重点项目、50件民生实事”，打造“10+1”工程的升级版。更加谋划长远。坚持“功成不必在我”，多干打基础、利长远的事，跑好接力棒，成就接力赛，不贪一时之功，不图一时之名，坚决不搞形式主义、政绩工程和浮夸之风，坚决不让今天的政绩成为明天的包袱。

各位代表，宜春已站上新的起点，前景广阔、任重道远。与其面对晚霞幻想，不如迎着朝阳苦干。让我们在市委的坚强领导下，凝心聚力，奋勇争先，为开启“五年决战同步小康”新征程而努力奋斗！
